# Association between harm reduction strategies and healthcare utilization in patients on long-term prescribed opioid therapy presenting to acute healthcare settings: a protocol for a systematic review and meta-analysis

**DOI:** 10.1186/s13643-019-0997-5

**Published:** 2019-04-05

**Authors:** Jean Deschamps, James Gilbertson, Sebastian Straube, Kathryn Dong, Frank P. MacMaster, Christina Korownyk, Lori Montgomery, Ryan Mahaffey, James Downar, Hance Clarke, John Muscedere, Katherine Rittenbach, Robin Featherstone, Meghan Sebastianski, Ben Vandermeer, Deborah Lynam, Ryan Magnussen, Sean M. Bagshaw, Oleksa G. Rewa

**Affiliations:** 1 Department of Critical Care Medicine, Faculty of Medicine and Dentistry, University of Alberta, 2-124E, Clinical Sciences Building, 8440-112 St, Edmonton, NW T6G 2B7 Canada; 2 School of Medicine, Faculty of Medicine and Dentistry, University of Alberta, Edmonton, Alberta Canada; 3 Division of Preventative Medicine, Department of Medicine, University of Alberta, 5-30 University Terrace, 8303–112 St. NW, Edmonton, Alberta T6G 2T4 Canada; 4 Department of Emergency Medicine, University of Alberta, 2J2.00 WC Mackenzie Health Sciences Centre, 8440 112 St. NW, Edmonton, Alberta T6G 2R7 Canada; 5 Departments of Psychiatry and Pediatrics, University of Calgary, Strategic Clinical Network for Addictions and Mental Health, 2888 Shaganappi Trail NW, Calgary, Alberta T3B 6A8 Canada; 6 Department of Family Medicine, University of Alberta, Suite 205 College Plaza, 8215 112 St. NW, Edmonton, Alberta T6G 2C8 Canada; 7 Department of Family Medicine, Calgary Chronic Pain Center, 1820 Richmond Road SW, Calgary, Alberta T2T 5C7 Canada; 8 Department of Anesthesia, University of Ottawa, Ottawa, Ontario Canada; 9 Department of Critical Care Medicine, University of Toronto, Toronto, Ontario Canada; 10 Department of Anesthesia and Pain Management, Toronto General Hospital, University Health Network, Toronto, Ontario Canada; 11 Transitional Pain Program, Toronto General Hospital, University Health Network, Toronto, Ontario Canada; 12 Department of Critical Care Medicine, Queen’s University, Kingston, Ontario Canada; 13 Addiction and Mental Health Strategic Clinical Network, Alberta Health Services, Edmonton, Alberta Canada; 14 Department of Psychiatry, University of Alberta, Edmonton, Alberta Canada; 15 Alberta Research Centre for Health Evidence, Department of Pediatrics, University of Alberta, Alberta,, Canada; 16 Alberta SPOR SUPPORT Unit KT Platform, Edmonton Clinical Health Academy, 4-486D, 11405–87 Avenue, Edmonton, Alberta T6G 1C9 Canada; 17 Knowledge Translation Platform, Alberta SPOR SUPPORT Unit Department of Pediatrics, University of Alberta, 362-B Heritage Medical Research Centre (HMRC), Alberta, Canada; 18 Primary Health Care Information Network, Edmonton, Alberta Canada; 19 Critical Care Strategic Clinical Network, Foothills Medical Centre, ICU Administration–Ground Floor, McCaig Tower, 3134 Hospital Drive, Calgary, Alberta T2N 2T9 Canada

**Keywords:** Narcotics, Addiction medicine, Substance-related disorders, Drug abuse, Hospital medicine

## Abstract

**Introduction:**

Opioids are routinely used to treat a variety of chronic conditions associated with pain. However, they are a class of medications with a significant potential for adverse health effects, with and without misuse. Opioid misuse, as defined as inappropriate use of appropriately prescribed opioids, is becoming more well-recognized publicly but does not have clear treatment options. Opioid misuse has been linked to variety of poor outcomes and its consequences have a significant impact on healthcare resource utilization. The evidence on harm reduction strategies to mitigate adverse events prompting presentation to acute care settings for patients presenting with long-term opioid use is sparse.

**Methods and analysis:**

We will perform a systematic review and meta-analysis to catalog effective harm reduction strategies and identify the most effective ones to reduce avoidable healthcare utilization in patients on long-term opioid therapy who present to acute health care settings with complications attributed to opioid misuse. A search strategy will be developed and executed by an information specialist; electronic databases (MEDLINE, EMBASE, CINAHL, Cochrane Library) and additional sources will be searched. Search themes will include opioids, chronic drug use, and acute healthcare settings. Citation screening, selection, quality assessment, and data abstraction will be performed in duplicate. A comprehensive inventory of harm reduction strategies will be developed. Data will be collected on patient-related outcomes associated with each identified harm reduction strategy. When sufficiently homogeneous data on interventions, population, and outcomes is available, it will be pooled for aggregate analysis. Evaluation of the methodological quality of individual studies and of the quality of the body of evidence will be performed. Our primary objective will be to identify harm reduction strategies that have been shown to result in clinically relevant and statistically significant improvements in patient outcomes and/or decreased healthcare utilization.

**Discussion:**

This study will better characterize harm reduction strategies for patients on long-term prescribed opioids presenting to acute healthcare settings. It will also add new knowledge and generate greater understanding of key knowledge gaps of the long-term prescribed opioid use and its impact on healthcare utilization.

**Systematic review registration:**

CRD42018088962.

**Electronic supplementary material:**

The online version of this article (10.1186/s13643-019-0997-5) contains supplementary material, which is available to authorized users.

## Introduction

### Description of the condition

The opioid crisis is the major public health problem facing Canada today. It has been deemed a public health emergency by both Health Canada and the US Surgeon General [1]. Opioid use is common in Canada, with over 21,523,000 prescriptions written in 2016, which equates to 595 opioid prescriptions per 1000 Canadians [2–4]. This is particularly alarming as prescribed opioids (i.e., fentanyl, hydromorphone, morphine, and oxycodone) account for more than half of all opioid poisoning hospitalization each year [2], and seniors account for a quarter of those, with more than half accidental in nature and with worse outcomes [1–4]. In the USA, over 60% of drug overdoses involve an opioid, and these most commonly involve opioids prescribed by healthcare practitioners [5]. Canada is second only to the USA regarding chronic opioid use, which led to the Canadian Centre on Substance Use and Addiction to publish a document addressing prescription drugs that are not only legal, have therapeutic uses, but also have a high potential for harm [6]. This document outlined five streams of action: prevention, education, treatment, monitoring, and enforcement [5]. Long-term opioid use is less recognized publicly, but has been linked to a variety of poor outcomes such as frequent hospitalizations, unintentional overdoses, and death [7, 8]. This is of particular importance with high-dose usage, which has recently come to be defined as greater than 90 morphine milligram equivalents (MME) daily [9]. The consequences of opioid-related healthcare visits have a significant impact on resource utilization, including emergency department (ED) presentations, hospital and intensive care unit (ICU) admissions, and significant patient morbidity and mortality as well as important socio-economic consequences [3, 10–13].

### Description of the intervention

Harm reduction strategies have been given multiple definitions in the literature. For the purposes of this review, harm reduction strategies are defined as “any policy or program designed to reduce drug-related harm without requiring the cessation of drug use; these interventions may be targeted at the individual, the family, community or society [14].” These may include, but are not limited to (1) in-hospital policies including medication review and referral to outpatient support programs; (2) outpatient measures, such as improving access to and use of prescription drug monitoring program, opioid agonist treatment programs, and referral to chronic pain centers; (3) physician education including web-based resources as well as workshops and seminars; and (4) policy development such as the creation of Alberta Health Services Harm Reduction Policy for Psychoactive Substance Use, the National Canadian Pain Guidelines, and the Canadian Federal Action on Opioids statement [5, 6, 14–20]. There is evidence of a modest reduction in mortality from these measures in the acute opioid toxicity setting [15]. However, the data available on the impact of harm reduction strategies in patients presenting with long-term prescribed opioid use that develop complications requiring presentation to acute care settings is sparse. The impact of such strategies may be substantial given the reported high risk of adverse events in these settings [11, 21–23]. Additionally, recent trends in opioid prescriptions show stability or growing prescription patterns and usage, despite policies and measures put in place to address this growing problem [24]. This highlights the complexity of outcomes associated with opioid usage, the potential for unknown interactions, and the need for further studies.

### Why is it important to do this review?

A better understanding of what factors lead to recurrent presentations to acute healthcare secondary to opioid misuse for patients who take daily prescribed opioids may enable more tailored management strategies for these patients, more appropriate follow-up planning at discharge from acute healthcare settings, improved resource allocation, and decreased acute healthcare presentations. This would in turn serve to alleviate the strain on emergency services, improve the capacity of emergency departments, and improve patient health and quality of life. The goal of this study is to explore patients with presumably appropriately prescribed opioids who suffer a complication related to their use leading to an acute healthcare setting presentation, secondary to either appropriate or inappropriate use. For the purpose of this study, opioid misuse will refer to the inappropriate use of opioids prescribed to the patient in question. We will not be focusing on illicit drug use as defined in this study by prescription opioids that are not prescribed to the patient in question or non-prescription opioids.

### Objectives

Among long-term prescribed opioid users presenting to acute care for reasons attributable to appropriate or inappropriate opioid use, we aim to identify the most effective harm reduction strategies to reduce avoidable health services use and improve outcomes.

## Methods and analysis

### Study design

We will perform a systematic review using the guidelines from Cochrane and the Centre for Reviews and Dissemination [25, 26], and reported according to the Preferred Reporting Items for Systematic review and Meta-analysis (PRISMA-P) Guideline (Additional file 1: Appendix 1) [27] and the Meta-analysis Of Observational Studies in Epidemiology (MOOSE) guidelines for observational studies (Additional file 1: Appendix 2) [28]. Data will be synthesized, and a meta-analysis will be conducted on aggregate data, if applicable.

### Study registration

The systematic review protocol has been registered with the PROSPERO (registration # CRD42018088962 on 2018/02/19) International Prospective Register of Systematic Reviews (https://www.crd.york.ac.uk/prospero/display_record.php?RecordID=88962).

### Ethics and dissemination

Data for this review will be sourced from available published and unpublished studies. As such, no patient-specific primary data will need to be collected, and formal health research ethics approval is not required. Our study will be disseminated through a conventional peer-reviewed publication and presentation at a scientific congress. In addition, we will disseminate our findings regionally and nationally through the Critical Care, Emergency Medicine and Addiction and Mental Health Alberta Health Strategic Clinical Networks and societies such as the Alberta Addiction and Mental Health Research Partnership Program, Canadian Critical Care Society, the Canadian Society of Addiction Medicine, the Canadian Centre on Substance Use and Addiction, and the Canadian Research Initiative in Substance Misuse, the Canadian Frailty Network, along with media sources, including Twitter and regional website platforms for research. If our protocol needs to be amended, the date, details of the change, and the rationale will be documented in the revised protocol and updated on PROSPERO.

### Criteria for considering studies for this review

#### Types of studies

We will consider all primary studies (i.e., randomized controlled trials, cohort studies, case-control studies, case series, and audits) and secondary analyses or evidence synthesis (i.e., systematic reviews, meta-analyses) that describe management and outcomes of patients in acute care healthcare settings who have been using prescribed opioids regularly. In addition, we will search reports from the National Information Center on Health Services Research and Health Care Technology (NICHSR) via the NICHSR ONESearch portal. We also will search study records via the trial registry platform ClinicalTrials.gov, guidelines via the National Guidelines Clearinghouse, and selected meeting abstracts from the past 3 years via the Conference Proceedings Citation Index database (Clarivate Analytics). We will include studies and abstracts only if they include sufficient detail for data analysis. We will include policy statements from relevant organizations and governmental agencies that include clinical practice guidelines and population-based statistics and data. We will review all guidelines for relevant evidence to inform our systematic review. The inclusion of all types of studies is based on the fact that some interventions/harm reduction strategies are amenable to randomized controlled trial design (i.e., coordinated care plan in emergency departments), while others may be assessed in a non-randomized fashion (i.e., state-wide policy statements). We will include only studies published in English or French. We will consider studies published after 1996 as this is when OxyContin was introduced and the current opioid epidemic is believed to have largely begun. We will exclude editorials, case reports, and narrative reviews.

#### Eligibility of individual studies

Studies will be included if they satisfy the following criteria, based on a PICO format question: studies with adults aged 18 years or older, on long-term or chronic opioid therapy (intending to reflect use of prescribed opioids more than 70% of days for at least 3 months [29] and that present to an acute healthcare setting secondary to a complication of prescribed opioid use; that include an intervention representing a harm reduction strategy, as defined as an attempt to decrease adverse consequences of long-term opioid use; that either compare the efficacy of the different interventions between each other or of individual interventions compared to current care; and that address patient or system related outcomes (e.g., ED visits, hospitalization, ICU admission, economic considerations).

#### Search methods

The search strategy will be developed and executed by an information specialist and will be peer-reviewed by a second research librarian (Table 1). The information specialist will search electronic databases: Ovid MEDLINE (1946–), Ovid EMBASE (1996–), Cumulative Index to Nursing and Allied Health Literature (CINAHL) via EBSCOhost (1937–), and Wiley Cochrane Library (inception–), including the Cochrane Database of Systematic Reviews and the Cochrane Central Register of Controlled Trials (CENTRAL). In addition, we will search reports from the National Information Center on Health Services Research and Health Care Technology (NICHSR) via the NICHSR ONESearch portal. We will also search study records via the trial registry platform ClinicalTrials.gov, guidelines via the National Guidelines Clearinghouse, and meeting abstracts via the Conference Proceedings Citation Index database (Clarivate Analytics). A combination of the following search themes will be used: (1) opioids; (2) long-term drug use; and (3) acute healthcare settings (emergency departments, acute care surgery, critical care, urgent care, and short-term inpatient stabilization). Results will be limited to human studies in adult populations, published since 1996 in English or French. We will additionally scan the reference list of relevant included studies for additional articles. Bibliographic records will be exported to an EndNote X9 (Clarivate Analytics, Philadelphia, Pennsylvania) database for screening, as defined in the studies eligibility section.

**Table 1:** Search strategy

Database: Ovid MEDLINE(R) Epub ahead of print, in-process, and other non-indexed citations, Ovid MEDLINE(R) Daily and Ovid MEDLINE(R) 1946 to present	
1	exp Narcotics/ (111132)
2	actiq*.tw,kf. (27)
3	carfentan*.tw,kf. (243)
4	codeine*.tw,kf. (4872)
5	demerol*.tw,kf. (231)
6	(dihydro-morph* or dihydromorph*).tw,kf. (451)
7	dilaudid*.tw,kf. (69)
8	dur?gesic*.tw,kf. (84)
9	fentanyl*.tw,kf. (16667)
10	fentora*.tw,kf. (9)
11	heroin.tw,kf. (12893)
12	(hydro-codone* or hydrocodone*).tw,kf. (858)
13	(hydro-morphone* or hydromorphone*).tw,kf. (1359)
14	morphine*.tw,kf. (47330)
15	narcotic*.tw,kf. (14412)
16	lorcet*.tw,kf. (5)
17	lortab*.tw,kf. (6)
18	opiate*.tw,kf. (23769)
19	opioid*.tw,kf. (73603)
20	(oxy-codone* or oxycodone*).tw,kf. (2670)
21	(oxy-contin* or oxycontin*).tw,kf. (226)
22	percocet*.tw,kf. (58)
23	percodan*.tw,kf. (14)
24	pethidine*.tw,kf. (2304)
25	phentanyl*.tw,kf. (119)
26	sublimaze*.tw,kf. (22)
27	vicodin*.tw,kf. (56)
28	or/1-27 [Combined MeSH & text words for opioids] (186296)
29	Addiction Medicine/ (4)
30	Behavior, Addictive/ (7744)
31	exp "Chemical and Drug Induced Liver Injury"/ (26678)
32	Drug abuse/ (87805)
33	exp Drug Misuse/ (10703)
34	Drug Overdose/ (9457)
35	Neurotoxicity Syndromes/ (4428)
36	exp Opioid-Related Disorders/ (22304)
37	Poisoning/ (21631)
38	Psychoses, Substance-Induced/ (5082)
39	Self-Injurious Behavior/ (6447)
40	Street Drugs/ae [adverse effects] (1421)
41	Substance-Related Disorders/ (87805)
42	Substance Withdrawal Syndrome/ (20325)
43	((abus* or addict* or chronic* or depend* or disorder* or intoxicat* or mis-us* or misus* or over-dos* or overdos* or poison* or withdrawal*) adj3 (drug* or fentanyl* or heroin* or narcotic* or opiate* or opioid* or oxy-co* or oxyco* or morphine*)).tw,kf. (97309)
44	((drug* or substance* or toxic*) adj2 psycho*).tw,kf. (18771)
45	or/29-44 [Combined MeSH & text words for chronic drug use] (263106)
46	Burn Units/ (2227)
47	Coronary Care Units/ (4202)
48	exp Critical Care/ (51242)
49	Critical Care Nursing/ (1223)
50	Emergency Medicine/ (11989)
51	Emergency Nursing/ (6602)
52	exp Perioperative Care/ and (acute* or critical* or emergenc* or intensiv* or trauma* or urgent*).mp. (19203)
53	Hospital Medicine/ (119)
54	exp Hospitals/ and (acute* or critical* or emergenc* or intensiv* or trauma* or urgent*).mp. (42779)
55	Hospitalization/ (91123)
56	Intensive Care Units/ (45436)
57	exp Life Support Care/ (8408)
58	Operating Rooms/ and (acute* or critical* or emergenc* or intensiv* or trauma* or urgent*).mp. (1581)
59	Respiratory Care Units/ (579)
60	exp Specialties, Surgical/ and (acute* or critical* or emergenc* or intensiv* or trauma* or urgent*).mp. (16202)
61	Surgery Department, Hospital/ and (acute* or critical* or emergenc* or intensiv* or trauma* or urgent*).mp. (1066)
62	((acute* or critical* or emergenc* or intensiv* or trauma* or urgent*) adj2 (care or centr* or department* or hospital* or unit* or ward*)).tw,kf. (270869)
63	((acute* or critical* or emergenc* or intensiv* or trauma* or urgent*) and (intraoperative or operative or perioperative or postoperative)).tw,kf. (114703)
64	((burn* or cardi* or coronary* or heart* or respiratory*) adj2 (care or department* or room* or unit* or ward*)).tw,kf. (27819)
65	ICU.tw,kf. (44322)
66	life support.tw,kf. (10639)
67	or/46-66 [Combined MeSH & text words for acute healthcare settings] (564082)
68	and/28,45,67 [Combined concepts for opioids, chronic drug use, & acute healthcare settings] (2136)
69	exp animals/ not humans/ (4426250)
70	68 not 69 [Exclude animal studies] (2116)
71	(adolescent/ or exp child/) not exp adult/ (1302784)
72	(adolescen* or child* or infan* or neonat* or p?ediatric* or youth).ti,jw. (1500398)
73	70 not (71 or 72) [Exclude pediatric studies] (1845)
74	(comment or editorial or news or newspaper article).pt. (1210379)
75	73 not 74 [Exclude opinion pieces] (1810)
76	("1996 *" or "1997 *" or "1998 *" or "1999 *" or 200* or 201*).dt. (17023135)
77	and/75-76 [date limit applied] (1410)
78	limit 77 to (english or french) (1322)
79	remove duplicates from 78 (1315)

### Study assessment

#### Study selection

Eligible articles will be identified through two phases, as defined in the studies eligibility section. In the first phase, two authors (JD and JG) will independently review the titles and abstracts of all retrieved articles and documents using EndNote X7 for potential inclusion. In the second phase, full texts of the selected articles will be retrieved, and two authors will independently review and select studies that meet the inclusion criteria. In each phase, disagreements will be resolved through discussion, and in the case of unresolved matters, a third author (OGR) will be involved. Reasons for exclusion of full text will be recorded and displayed in a PRISMA diagram format.

#### Data extraction

For full-text studies selected for inclusion, relevant information will be abstracted using piloted and standardized electronic data forms independently by the same two authors. Descriptive analysis of the articles will be performed by each author individually and will be used to write a narrative review of the identified harm reduction strategies, their benefits and risks, the studied outcomes, and the quality of the studies’ conduction and reporting. Abstracted data will be then compared amongst the two authors. Disagreements will be resolved through discussion. In the case of unresolved matters, a third author (OGR) will be involved.

Data extracted will include harm reductions strategies identified from included articles and documents, study features, patient characteristics (i.e., age, sex, comorbid disease, organ failure scores, acuity of illness scores, case-mix and diagnostic classification, respiratory status and mechanical ventilation), and outcome data, including alteration in opioid usage, adverse events, hospital and ICU length of stay, number of presentations to emergency departments, number of readmissions to hospitals and ICUs, time to next presentation to the emergency departments, time to first readmission to hospital and ICU, survival at 28 days, 6 months, and 1 year, and quality of life questionnaires.

#### Method for missing data

We will contact the study authors for any relevant missing data. For continuous outcomes, standard deviations may be imputed if they are not either reported or calculable from other given values within the study.

#### Quality assessment of studies

The methodological quality of each study will be independently analyzed by two authors (JD and JG) using the Newcastle-Ottawa Quality Assessment Scale (NOS) for observational studies (Additional file 1: Appendix 3) [30] and the Cochrane Risk of Bias Tool for randomized controlled trials (Additional file 1: Appendix 4) [31]. Disagreements will be resolved through discussion. In the case of unresolved matters, a third author (OGR) will be involved. We will assess the quality of the body of evidence using the Grading of Recommendations Assessment, Development and Evaluation (GRADE) [32, 33].

#### Data analysis and synthesis

A comprehensive inventory of harm reduction strategies will be developed from the included studies. Amongst same study types (i.e., randomized controlled trials, cohort studies, case-control studies), when interventions, populations, and outcomes are considered sufficiently clinically homogenous, data will be pooled using a DerSimonian Laird random-effects model [34]. Binary outcomes (e.g., hospital admission) will be pooled using unadjusted or adjusted risk ratios as appropriate; odds ratios and adjusted results may be used if data from the studies is insufficient for risk ratios calculation. Continuous data (e.g., hospital length of stay) will be pooled using mean differences or standardized mean differences—unadjusted if possible. Statistical heterogeneity will be assessed using the *I*^2^ statistic [35]. If any meta-analysis contains more than 10 studies we will assess small study bias visually using a funnel plot and statistically using Egger’s test [36].

#### Study comparison and assessment

The primary objective of the study will be a simple identification of harm reduction strategies currently in use for patients presenting to healthcare settings with complications related to prescribed opioid use. The different studies will either compare interventions between themselves in different arms or more commonly will compare an intervention to established standard of care or usual care in the institution for the relevant complication or opioid use. As such, if possible, a quantitative comparison based on identified outcomes listed in sections above will be performed to establish which interventions are useful, not useful, or preferable to others. This will be performed from meta-analyses from randomized-controlled trials and non-randomized-controlled trials separately, and the results will be discussed separately as they apply to each study design. If a quantitative comparison is not possible, a qualitative comparison based on the strength of supporting data will be performed to help identify the most impactful or evidence-based strategies.

## Discussion

### General

Opioid use is an evolving problem which is better understood and publicized in the setting of acute intoxication, and for which long-term opioid use and its consequences represent the less well-recognized part. Long-term opioid use has high prevalence, and while the adverse health outcomes associated with it may have a relatively low incidence, they will still be commonly encountered. These adverse health outcomes include hospital and ICU length of stay, number of presentations and time to next presentation to emergency departments, number of readmissions to hospitals and ICUs, time to first readmission to hospital and ICU, unexpected death, and increased health resources in the emergency department, acute medical care, and ICU. The use of opioids has been shown to lead to poor patient outcomes in terms of increased mortality, and from a public health perspective to significant healthcare resource utilization and societal long-term costs in terms of work absenteeism and loss of productivity [1, 2, 13]. This likely extends to increased resource utilization in acute healthcare, although the precise impact and solutions have not been well studied or defined. However, it has been noted that patients with acute intoxication and with long-term opioid use (whether or not admitted primarily from opioid use complications or not) have an incremental risk for hospitalization and ICU admission compared to the general population [1, 2, 10, 11, 37]. Additionally, patients with prior opioid use have longer hospitalization, ICU length of stay, mechanical ventilation, higher opioid needs, and associated complications as compared to general population [1, 2, 10–13, 23, 37–40]. The specific subset of patients with long-term prescribed opioid use is less well studied but may have similar outcomes [22, 41]. Part of these worse outcomes may be related to poor knowledge by healthcare providers at all levels on how to properly choose and adjust opioids [19, 20] and how to properly control pain and drug withdrawal symptoms.

Harm reduction strategies have been well studied in emergency department, medical wards, and ICUs, and may include elements such as medication list review on admission and discharge, presence of a pharmacist during rounds [14, 19], physician education for appropriate opioid dosing [19, 20], and awareness of drug withdrawal symptoms. It may also relate to strategies initiated in the acute care settings that extend in the outpatient setting such as better identification processes for opioid use, progression to opioid use disorder, and organized process for referral to appropriate outpatient counseling and treatment program, pain clinics, and transmission of information to general practitioners. Our study will characterize these features in an acute care setting, add new knowledge, and generate greater understanding of key knowledge gaps for long-term prescribed opioid use and its impact on healthcare, as well as related harm reduction strategies. We expect this review will contribute to evidence-based recommendations for inpatient and outpatient management of long-term prescribed opioid users, and thus improved outcomes and decreased healthcare resource utilization. This study may also help identify measures designed to enhance the flow of communication between acute care providers and community healthcare providers related to the management of patients with long-term prescribed opioid use who are at risk for opioid use disorder and abuse.

### Expected limitations

There are a number of potential limitations for this review. First, long-term prescribed opioid use complications may not be well recognized by healthcare professionals and captured by studies, particularly in the acute care settings, because many complications (such as falls, fractures, and accidents) are not identified as resulting from opioid use [42]. Additionally, progression to opioid misuse is inherently difficult to identify because of the underlying need for opioids in most of these patients [42–46]. As such, there is probably a limited amount of available studies in this specific setting, which will limit our ability to identify harm reduction strategies and their outcomes. We will use a broad search strategy to capture as many relevant studies as possible. Second, current harm reduction strategies are often highly dependent on patient cooperation, which can be difficult during acute illness, and which also might limit their applicability both in acute and outpatient healthcare settings. Specific interventions may be more applicable than others, and their effectiveness will need to be assessed on an individual basis. Third, it is anticipated that due to the paucity of focused literature on harm reduction with long-term opioid use in acute healthcare settings, and the expected considerable heterogeneity across studies in terms of methodology and patient characteristics, an aggregate analysis of the harm reductions in the form of a meta-analysis may not be feasible. Fourth, we will limit the study to sources in French and English language, which may introduce a language bias and limit the evidence available for harm reduction strategies relating to certain populations; however, it has been previously shown that there is no evidence of a systematic bias from the use of language restrictions in systematic review-based meta-analyses [47], which will limit this issue. Nevertheless, a systematic appreciation of the data available will be important in shaping future research and giving direction for healthcare practitioners providing care for patients using opioids in the long term and recovering from acute illness.

## Additional file

Additional file 1: Assessment scales and guidelines used for the systematic review and meta-analysis. (PDF 2225 kb)
