# Supplementary material for: Association between harm reduction strategies and healthcare utilization in patients on long-term prescribed opioid therapy presenting to acute healthcare settings: a protocol for a systematic review and meta-analysis
Source: Syst Rev. 2019 Apr 5;8:88. doi: 10.1186/s13643-019-0997-5 (PMC6449896; doi:10.1186/s13643-019-0997-5)
Supplement: Supplementary file 1 — Assessment scales and guidelines used for the systematic review and meta-analysis. (PDF 2225 kb) [file 13643_2019_997_MOESM1_ESM.pdf]

Appendix 1: Preferred Reporting Items for Systematic review and Meta-Analysis Guideline (24)

PRISMA-P (Preferred Reporting Items for Systematic review and Meta-Analysis Protocols) 2015 checklist: recommended items to address in a systematic review protocol*

Section and topic	Item No	Checklist item
**ADMINISTRATIVE INFORMATION**		
Title:		
Identification	1a	Identify the report as a protocol of a systematic review
Update	1b	If the protocol is for an update of a previous systematic review, identify as such
Registration	2	If registered, provide the name of the registry (such as PROSPERO) and registration number
Authors:		
Contact	3a	Provide name, institutional affiliation, e-mail address of all protocol authors; provide physical mailing address of corresponding author
Contributions	3b	Describe contributions of protocol authors and identify the guarantor of the review
Amendments	4	If the protocol represents an amendment of a previously completed or published protocol, identify as such and list changes; otherwise, state plan for documenting important protocol amendments
Support:		
Sources	5a	Indicate sources of financial or other support for the review
Sponsor	5b	Provide name for the review funder and/or sponsor
Role of sponsor or funder	5c	Describe roles of funder(s), sponsor(s), and/or institution(s), if any, in developing the protocol
**INTRODUCTION**		
Rationale	6	Describe the rationale for the review in the context of what is already known
Objectives	7	Provide an explicit statement of the question(s) the review will address with reference to participants, interventions, comparators, and outcomes (PICO)
**METHODS**		
Eligibility criteria	8	Specify the study characteristics (such as PICO, study design, setting, time frame) and report characteristics (such as years considered, language, publication status) to be used as criteria for eligibility for the review
Information sources	9	Describe all intended information sources (such as electronic databases, contact with study authors, trial registers or other grey literature sources) with planned dates of coverage
Search strategy	10	Present draft of search strategy to be used for at least one electronic database, including planned limits, such that it could be repeated
Study records:		
Data management	11a	Describe the mechanism(s) that will be used to manage records and data throughout the review

Appendix 2: Meta-analysis Of Observational Studies in Epidemiology guidelines for observational studies (25)

Item No	Recommendation	Reported on Page No
Reporting of background should include		
1	Problem definition	4
2	Hypothesis statement	-
3	Description of study outcome(s)	7
4	Type of exposure or intervention used	4-6
5	Type of study designs used	5-7
6	Study population	6
Reporting of search strategy should include		
7	Qualifications of searchers (eg, librarians and investigators)	5, Title page
8	Search strategy, including time period included in the synthesis and key words	5, Table 1
9	Effort to include all available studies, including contact with authors	6
10	Databases and registries searched	6
11	Search software used, name and version, including special features used (eg, explosion)	6
12	Use of hand searching (eg, reference lists of obtained articles)	6
13	List of citations located and those excluded, including justification	8, Table 2, Fig 1
14	Method of addressing articles published in languages other than English	-
15	Method of handling abstracts and unpublished studies	6
16	Description of any contact with authors	6
Reporting of methods should include		
17	Description of relevance or appropriateness of studies assembled for assessing the hypothesis to be tested	6-8
18	Rationale for the selection and coding of data (eg, sound clinical principles or convenience)	6-8
19	Documentation of how data were classified and coded (eg, multiple raters, blinding and interrater reliability)	6-8
20	Assessment of confounding (eg, comparability of cases and controls in studies where appropriate)	7
21	Assessment of study quality, including blinding of quality assessors, stratification or regression on possible predictors of study results	6-7
22	Assessment of heterogeneity	7
23	Description of statistical methods (eg, complete description of fixed or random effects models, justification of whether the chosen models account for predictors of study results, dose-response models, or cumulative meta-analysis) in sufficient detail to be replicated	7-8

24	Provision of appropriate tables and graphics	Tables 2-7, Figs 2-7
Reporting of results should include		
25	Graphic summarizing individual study estimates and overall estimate	Figs 3-7
26	Table giving descriptive information for each study included	Table 2
27	Results of sensitivity testing (eg, subgroup analysis)	Fig 3, Table 3
28	Indication of statistical uncertainty of findings	12-16

Item No	Recommendation	Reported on Page No
Reporting of discussion should include		
29	Quantitative assessment of bias (eg, publication bias)	12, Fig 2
30	Justification for exclusion (eg, exclusion of non-English language citations)	6
31	Assessment of quality of included studies	6-7
Reporting of conclusions should include		
32	Consideration of alternative explanations for observed results	17-19
33	Generalization of the conclusions (ie, appropriate for the data presented and within the domain of the literature review)	20
34	Guidelines for future research	-
35	Disclosure of funding source	20

From: Stroup DF, Berlin JA, Morton SC, et al, for the Meta-analysis Of Observational Studies in Epidemiology (MOOSE) Group. Meta-analysis of Observational Studies in Epidemiology. A Proposal for Reporting. *JAMA*. 2000;283(15):2008-2012. doi: 10.1001/jama.283.15.2008.

Appendix 3: Newcastle-Ottawa Quality Assessment Scale for observational study (30)

CODING MANUAL FOR CASE-CONTROL STUDIES

SELECTION

1) **Is the Case Definition Adequate?**
   a) Requires some independent validation (e.g. >1 person/record/time/process to extract information, or reference to primary record source such as x-rays or medical/hospital records)
   b) Record linkage (e.g. ICD codes in database) or self-report with no reference to primary record ☆
   c) No description

2) **Representativeness of the Cases**
   a) All eligible cases with outcome of interest over a defined period of time, all cases in a defined catchment area, all cases in a defined hospital or clinic, group of hospitals, health maintenance organisation, or an appropriate sample of those cases (e.g. random sample)
   b) Not satisfying requirements in part (a), or not stated. ☆

3) **Selection of Controls**
   This item assesses whether the control series used in the study is derived from the same population as the cases and essentially would have been cases had the outcome been present.
   a) Community controls (i.e. same community as cases and would be cases if had outcome) ☆
   b) Hospital controls, within same community as cases (i.e. not another city) but derived from a hospitalised population
   c) No description

4) **Definition of Controls**
   a) If cases are first occurrence of outcome, then it must explicitly state that controls have no history of this outcome. If cases have new (not necessarily first) occurrence of outcome, then controls with previous occurrences of outcome of interest should not be excluded.
   b) No mention of history of outcome ☆

COMPARABILITY

1) **Comparability of Cases and Controls on the Basis of the Design or Analysis**

A maximum of 2 stars can be allotted in this category

Either cases and controls must be matched in the design and/or confounders must be adjusted for in the analysis. Statements of no differences between groups or that differences were not statistically significant are not sufficient for establishing comparability. Note: If the odds ratio for the exposure of interest is adjusted for the confounders listed, then the groups will be considered to be comparable on each variable used in the adjustment.

There may be multiple ratings for this item for different categories of exposure (e.g. ever vs. never, current vs. previous or never)

Age = ☆ , Other controlled factors = ☆

EXPOSURE

1) **Ascertainment of Exposure**

Allocation of stars as per rating sheet

2) Non-Response Rate
Allocation of stars as per rating sheet

CODING MANUAL FOR COHORT STUDIES

SELECTION

1) Representativeness of the Exposed Cohort

Item is assessing the representativeness of exposed individuals in the community, not the representativeness of the sample of women from some general population. For example, subjects derived from groups likely to contain middle class, better educated, health oriented women are likely to be representative of postmenopausal estrogen users while they are not representative of all women (e.g. members of a health maintenance organisation (HMO) will be a representative sample of estrogen users. While the HMO may have an under-representation of ethnic groups, the poor, and poorly educated, these excluded groups are not the predominant users users of estrogen).

Allocation of stars as per rating sheet

2) Selection of the Non-Exposed Cohort
Allocation of stars as per rating sheet

3) Ascertainment of Exposure
Allocation of stars as per rating sheet

4) Demonstration That Outcome of Interest Was Not Present at Start of Study
In the case of mortality studies, outcome of interest is still the presence of a disease/ incident, rather than death. That is to say that a statement of no history of disease or incident earns a star.

COMPARABILITY

1) Comparability of Cohorts on the Basis of the Design or Analysis
A maximum of 2 stars can be allotted in this category
Either exposed and non-exposed individuals must be matched in the design and/or confounders must be adjusted for in the analysis. Statements of no differences between groups or that differences were not statistically significant are not sufficient for establishing comparability. Note: If the relative risk for the exposure of interest is adjusted for the confounders listed, then the groups will be considered to be comparable on each variable used in the adjustment.
There may be multiple ratings for this item for different categories of exposure (e.g. ever vs. never, current vs. previous or never)
Age = ☆Other controlled factors = ☆

OUTCOME

1) Assessment of Outcome

For some outcomes (e.g. fractured hip), reference to the medical record is sufficient to satisfy the requirement for confirmation of the fracture. This would not be adequate for vertebral fracture outcomes where reference to x-rays would be required.

a) Independent or blind assessment stated in the paper, or confirmation of the outcome by reference to secure records (x-rays, medical records, etc.) ☆
b) Record linkage (e.g. identified through ICD codes on database records) ☆
c) Self-report (i.e. no reference to original medical records or x-rays to confirm the outcome)
d) No description.

2) Was Follow-Up Long Enough for Outcomes to Occur

An acceptable length of time should be decided before quality assessment begins (e.g. 5 yrs. for exposure to breast implants)

3) Adequacy of Follow Up of Cohorts

This item assesses the follow-up of the exposed and non-exposed cohorts to ensure that losses are not related to either the exposure or the outcome.

Allocation of stars as per rating sheet

Appendix 4: Cochrane Risk of Bias Tool for Randomized Controlled Trials (31)

Cochrane Risk of Bias Tool for Randomized Controlled Trials

**RANDOM SEQUENCE GENERATION**<br>**Selection bias (biased allocation to interventions) due to inadequate generation of a randomised sequence.**	
Criteria for a judgment of 'Low risk' of bias.	The investigators describe a random component in the sequence generation process such as:<br>• Referring to a random number table;<br>• Using a computer random number generator;<br>• Coin tossing;<br>• Shuffling cards or envelopes;<br>• Throwing dice;<br>• Drawing of lots;<br>• Minimization*.<br>*Minimization may be implemented without a random element, and this is considered to be equivalent to being random.
Criteria for the judgment of 'High risk' of bias.	The investigators describe a non-random component in the sequence generation process. Usually, the description would involve some systematic, non-random approach, for example:<br>• Sequence generated by odd or even date of birth;<br>• Sequence generated by some rule based on date (or day) of admission;<br>• Sequence generated by some rule based on hospital or clinic record number.<br>Other non-random approaches happen much less frequently than the systematic approaches mentioned above and tend to be obvious. They usually involve judgement or some method of non-random categorization of participants, for example:<br>• Allocation by judgement of the clinician;<br>• Allocation by preference of the participant;<br>• Allocation based on the results of a laboratory test or a series of tests;<br>• Allocation by availability of the intervention.
Criteria for the judgment of 'Unclear risk' of bias.	Insufficient information about the sequence generation process to permit judgement of 'Low risk' or 'High risk'.

<table>
<tr><td colspan="2">ALLOCATION CONCEALMENT
Selection bias (biased allocation to interventions) due to inadequate concealment of allocations prior to assignment.</td></tr>
<tr><td>Criteria for a judgment of ‘Low risk’ of bias.</td><td>Participants and investigators enrolling participants could not foresee assignment because one of the following, or an equivalent method, was used to conceal allocation:
• Central allocation (including telephone, web-based and pharmacy-controlled randomization);
• Sequentially numbered drug containers of identical appearance;
• Sequentially numbered, opaque, sealed envelopes.</td></tr>
<tr><td>Criteria for the judgment of ‘High risk’ of bias.</td><td>Participants or investigators enrolling participants could possibly foresee assignments and thus introduce selection bias, such as allocation based on:
• Using an open random allocation schedule (e.g. a list of random numbers);
• Assignment envelopes were used without appropriate safeguards (e.g. if envelopes were unsealed or non-opaque or not sequentially numbered);
• Alternation or rotation;
• Date of birth;
• Case record number;
• Any other explicitly unconcealed procedure.</td></tr>
<tr><td>Criteria for the judgment of ‘Unclear risk’ of bias.</td><td>Insufficient information to permit judgement of ‘Low risk’ or ‘High risk’. This is usually the case if the method of concealment is not described or not described in sufficient detail to allow a definite judgement – for example if the use of assignment envelopes is described, but it remains unclear whether envelopes were sequentially numbered, opaque and sealed.</td></tr>
<tr><td colspan="2">SELECTIVE REPORTING
Reporting bias due to selective outcome reporting.</td></tr>
<tr><td>Criteria for a judgment of ‘Low risk’ of bias.</td><td>Any of the following:
• The study protocol is available and all of the study’s pre-specified (primary and secondary) outcomes that are of interest in the review have been reported in the pre-specified way;
• The study protocol is not available but it is clear that the published reports include all expected outcomes, including those that were pre-specified (convincing text of this nature may be uncommon).</td></tr>
<tr><td>Criteria for the judgment of ‘High risk’ of bias.</td><td>Any one of the following:
• Not all of the study’s pre-specified primary outcomes have been reported;
• One or more primary outcomes is reported using measurements, analysis methods or subsets of the data (e.g. subscales) that were not pre-specified;
• One or more reported primary outcomes were not pre-specified</td></tr>
</table>

	(unless clear justification for their reporting is provided, such as an unexpected adverse effect);<br>• One or more outcomes of interest in the review are reported incompletely so that they cannot be entered in a meta-analysis;<br>• The study report fails to include results for a key outcome that would be expected to have been reported for such a study.
Criteria for the judgment of 'Unclear risk' of bias.	Insufficient information to permit judgement of 'Low risk' or 'High risk'. It is likely that the majority of studies will fall into this category.

**OTHER BIAS**<br>**Bias due to problems not covered elsewhere in the table.**	
Criteria for a judgment of 'Low risk' of bias.	The study appears to be free of other sources of bias.
Criteria for the judgment of 'High risk' of bias.	There is at least one important risk of bias. For example, the study:<br>• Had a potential source of bias related to the specific study design used; or<br>• Has been claimed to have been fraudulent; or<br>• Had some other problem.
Criteria for the judgment of 'Unclear risk' of bias.	There may be a risk of bias, but there is either:<br>• Insufficient information to assess whether an important risk of bias exists; or<br>• Insufficient rationale or evidence that an identified problem will introduce bias.

**BLINDING OF PARTICIPANTS AND PERSONNEL**<br>**Performance bias due to knowledge of the allocated interventions by participants and personnel during the study.**	
Criteria for a judgment of 'Low risk' of bias.	Any one of the following:<br>• No blinding or incomplete blinding, but the review authors judge that the outcome is not likely to be influenced by lack of blinding;<br>• Blinding of participants and key study personnel ensured, and unlikely that the blinding could have been broken.
Criteria for the judgment of 'High risk' of bias.	Any one of the following:<br>• No blinding or incomplete blinding, and the outcome is likely to be influenced by lack of blinding;<br>• Blinding of key study participants and personnel attempted, but likely that the blinding could have been broken, and the outcome is likely to be influenced by lack of blinding.
Criteria for the judgment of 'Unclear risk' of bias.	Any one of the following:<br>• Insufficient information to permit judgment of 'Low risk' or 'High risk';<br>• The study did not address this outcome.

BLINDING OF OUTCOME ASSESSMENT<br>**Detection bias due to knowledge of the allocated interventions by outcome assessors.**	
Criteria for a judgment of ‘Low risk’ of bias.	Any one of the following:<br>• No blinding of outcome assessment, but the review authors judge that the outcome measurement is not likely to be influenced by lack of blinding;<br>• Blinding of outcome assessment ensured, and unlikely that the blinding could have been broken.
Criteria for the judgment of ‘High risk’ of bias.	Any one of the following:<br>• No blinding of outcome assessment, and the outcome measurement is likely to be influenced by lack of blinding;<br>• Blinding of outcome assessment, but likely that the blinding could have been broken, and the outcome measurement is likely to be influenced by lack of blinding.
Criteria for the judgment of ‘Unclear risk’ of bias.	Any one of the following:<br>• Insufficient information to permit judgment of ‘Low risk’ or ‘High risk’;<br>• The study did not address this outcome.
**INCOMPLETE OUTCOME DATA**<br>**Attrition bias due to amount, nature or handling of incomplete outcome data.**	
Criteria for a judgment of ‘Low risk’ of bias.	Any one of the following:<br>• No missing outcome data;<br>• Reasons for missing outcome data unlikely to be related to true outcome (for survival data, censoring unlikely to be introducing bias);<br>• Missing outcome data balanced in numbers across intervention groups, with similar reasons for missing data across groups;<br>• For dichotomous outcome data, the proportion of missing outcomes compared with observed event risk not enough to have a clinically relevant impact on the intervention effect estimate;<br>• For continuous outcome data, plausible effect size (difference in means or standardized difference in means) among missing outcomes not enough to have a clinically relevant impact on observed effect size;<br>• Missing data have been imputed using appropriate methods.
Criteria for the judgment of ‘High risk’ of bias.	Any one of the following:<br>• Reason for missing outcome data likely to be related to true outcome, with either imbalance in numbers or reasons for missing data across intervention groups;<br>• For dichotomous outcome data, the proportion of missing outcomes compared with observed event risk enough to induce clinically relevant bias in intervention effect estimate;

	• For continuous outcome data, plausible effect size (difference in means or standardized difference in means) among missing outcomes enough to induce clinically relevant bias in observed effect size;<br>• 'As-treated' analysis done with substantial departure of the intervention received from that assigned at randomization;<br>• Potentially inappropriate application of simple imputation.
Criteria for the judgment of 'Unclear risk' of bias.	Any one of the following:<br>• Insufficient reporting of attrition/exclusions to permit judgement of 'Low risk' or 'High risk' (e.g. number randomized not stated, no reasons for missing data provided);<br>• The study did not address this outcome.

Thresholds for Converting the Cochrane Risk of Bias Tool to AHRQ Standards (Good, Fair, and Poor)

Good quality: All criteria met (i.e. low for each domain)

Using the Cochrane ROB tool, it is possible for a criterion to be met even when the element was technically not part of the method. For instance, a judgment that knowledge of the allocated interventions was adequately prevented can be made even if the study was not blinded, if EPC team members judge that the outcome and the outcome measurement are not likely to be influenced by lack of blinding.

Fair quality: One criterion not met (i.e. high risk of bias for one domain) or two criteria unclear, and the assessment that this was **unlikely** to have biased the outcome, and there is no known important limitation that could invalidate the results

Poor quality: One criterion not met (i.e. high risk of bias for one domain) or two criteria unclear, and the assessment that this was **likely** to have biased the outcome, and there are important limitations that could invalidate the results

Poor quality: Two or more criteria listed as high or unclear risk of bias
